# The Effect of Sensor Dimensions on the Performance of Flexible Hot Film Shear Stress Sensors

**DOI:** 10.3390/mi10050305

**Published:** 2019-05-06

**Authors:** Jin-Jin Wang, Hong Hu, Chao-Zhan Chen

**Affiliations:** School of Mechanical Engineering and Automation, Harbin Institute of Technology, Shenzhen, Guangdong 518055, China; jinjinking@outlook.com (J.-J.W.); chaozhanchen@aliyun.com (C.-Z.C.)

**Keywords:** flexible substrate, nickel thermistor, hot film sensor, temperature coefficient of resistance (TCR), time constant, sensitivity

## Abstract

This paper presents a study to determine the effect of sensor dimensions (length, width, and thickness) on the performance of flexible hot film shear stress sensors. The sensing component of a hot film sensor is nickel thermistor, and the flexible substrate material is polyimide. Several groups of flexible hot film shear stress sensors with different lengths, widths, and thicknesses were studied. The temperature coefficient of resistance (TCR) was measured. The TCR increased slightly with increasing thickness. The frequency response (time constant) of the flexible hot film shear stress sensor was obtained by the square wave, while the sensitivity was tested in a wind tunnel. The study found that as the sensor length was shortened, the frequency response increased, and the sensitivity decreased.

## 1. Introduction

**Figure 1:**
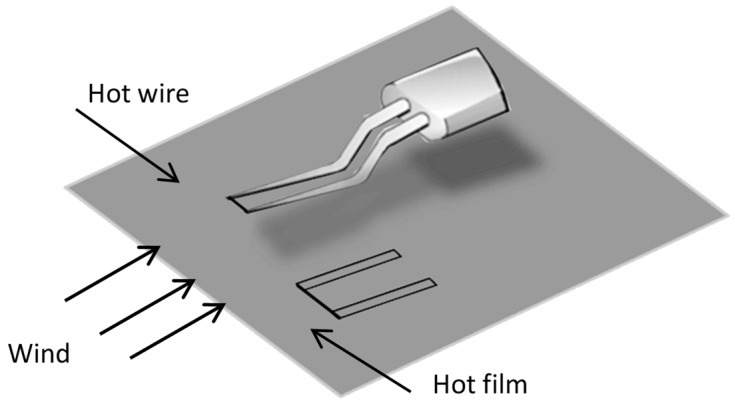
Hot wire and hot film used in the wind tunnel.

When measuring boundary layer in a wind tunnel, hot wires have always been used. The hot wire is always a tungsten wire. The two ends of the tungsten wire are welded on a support. Figure 1 shows the hot wire and flexible hot film installed on a flat plate. 

The hot wire is easy to break because the tungsten wire is hung in the flow with just two ends to support it. Due to the limitation of the support, it cannot be very close to the wall, which means that it cannot detect the thin boundary layer at a high Reynolds number [1]. To overcome these problems, a flexible hot film shear stress sensor that can directly stick to the wall becomes desirable [2]. The hot film deposited on the flexible substrate has a greater mechanical strength than the hot wire. Since the thickness of the sensor is in micron-dimension, the boundary layer at a high Reynolds number can be measured, and the fluid will not be disturbed. 

Recently, the flexible hot film shear stress sensor has drawn attention. This sensor can be curved freely and adhered to complicated surfaces, such as intelligent skin and wearable devices [3]. The fabrication process of flexible substrate sensors are simpler than for rigid substrates [4]. The flexible hot film shear stress sensors also have a lower thermal inertia and quicker response than those of conventional rigid sensors [5].

In our previous research, a flexible hot film shear stress sensor has been developed [6]. The characteristics of the sensor are tested. Performance of the flexible hot film sensor, especially the frequency response, is degraded from that of the hot wire [7]. There are many factors influencing the performance of flexible hot film shear stress sensors, such as thermistor and substrate properties [5,8,9] and annealing treatment [6,10]. Sensor size also has an effect on the performance of the shear stress sensor [11]. J. B. Huang has shown that the sensitivity is length dependent and the width of the sensor has a minor effect on the sensitivity [12]. However, as the thermistor they used was polysilicon, questions remain about the nickel thermistor. M. J. Moen reported that frequency response increased and sensitivity decreased with the decreasing of the sensor size [13]. However, as the nickel thermistor with the same aspect ratio was on a glass substrate, the situation may be different if the nickel thermistor was on a polyimide substrate. With regard to the latter, J. Z. Ren found that small thermistor dimensions are helpful for increasing frequency response, and that an increase of film thickness benefits a higher temperature coefficient of resistance (TCR) [14]. However, the detailed sensor size (length, width, and thickness) effect on the performance of a flexible shear stress sensor was not reported. In order to optimize the sensor design and improve its performance, the effect of size on the flexible hot film urgently needs to be understood.

In this paper, an experiment was undertaken to investigate the sensor size effect on the performance of the flexible hot film shear stress sensor. Several groups of nickel thermistors on a polyimide substrate were used for an experimental study to determine the effects of different length, width, and thickness on the TCR, response speed, and sensitivity. The flexible hot film shear stress sensor’s performance can be improved through optimizing the sensor design. 

## 2. Flexible Hot Film Shear Stress Sensor and its Performance

### 2.1. Structure and Fabrication

**Figure 2:**
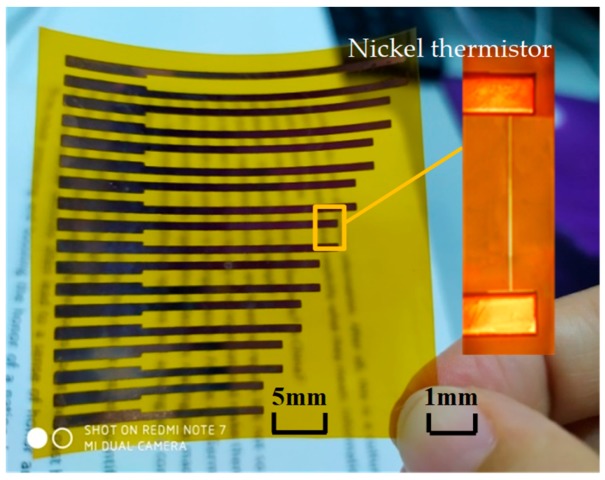
The sensing element of flexible hot film shear stress sensor.

The flexible hot film shear stress sensor fabricated is displayed in Figure 2. The nickel thermistor serves as the sensing element, which has a size of 2 mm × 0.01 mm × 0.0003 mm. The copper wires connect the sensing element with the outer circuit. The flexible substrate is a ready-made polyimide film with a thickness of 50 μm. The fabrication process is improved based on our previous technology [6] and was as follows: the polyimide was prepared by ultrasonic cleaning; then the nickel thermistor was obtained by a lift-off technique, in which a positive photoresist AZ1500 was used to pattern before the nickel film was deposited on the substrate by magnetron sputtering. The lift-off technique avoids the etching failure in the usual photolithography process and significantly improves the Ni thermistor. There was no interlayer, such as Cr and Ti, between the film and substrate; lastly, the copper wires were sputtered using a stencil instead of complicated photolithography procedures. Compared with the accuracy of the copper wire dimensions, the adhesion strength between the Cu wires and the polyimide (PI) film is much more important and will be better by using a stencil instead of photolithography.

### 2.2. Principle and Performance

The flexible hot film shear stress sensor works on the basis of the temperature-resistance characteristic of nickel thermistor, that is, the nickel thermistor’s resistance changes when the temperature changes. The temperature coefficient of resistance (TCR) is defined as the increase of resistance R by percentage for one degree increase of temperature T. R changes by dR when T changes by dT, $R_{0}$ is the cold resistance of the flexible hot film shear stress sensor at room temperature $T_{0}$. TCR can be written as follows:

(1) $$\mathrm{TCR}=\alpha =\frac{dR}{dT}\cdot \frac{1}{R}=\frac{R-R_{0}}{\left(T-T_{0}\right)\cdot R_{0}}$$

where *α* is TCR, reported in ppm/°C. From Equation (1), R can be calculated when the temperature changes.

When a fluid with a certain velocity flows through the flexible hot film shear stress sensor, it will take heat away from the thermistor, and then the temperature of the thermistor changes. As a result of the thermistor’s temperature-resistance characteristic, the resistance of the thermistor will change accordingly. The heat loss is related to the shear stress. Based on the energy balance theory, the heating energy arising from the thermistor equals the energy loss to the ambient flow and substrate. The convective transfer of heat from the thermistor to the ambient flow is a function of the flow velocity. The heating power P and the shear stress $\tau_{w}$, follow the relation:

(2) $$P=\left(T_{t}-T_{f}\right)\left(A+B\tau_{w}^{1/3}\right)$$

where $T_{t}$ and $T_{f}$ are the temperature of the thermistor and the flow, both reported in K; P is reported in W; $\tau_{w}$ is reported in Pa; A and B are calibration constants.

When the flexible hot film shear stress sensor works in the circuit, the heating power P can be written as:

(3) $$P=E^{2}/R$$

where E is the voltage across the sensor, in V. So, Equation (2) becomes:

(4) $$E^{2}=R\left(T_{t}-T_{f}\right)\left(A+B\tau_{w}^{1/3}\right)$$

After calibration, from Equations (1) and (4), the wall shear stress $\tau_{w}$ can be inferred.

The flexible hot film shear stress sensor is operated in constant temperature (CT) mode when calibrating the constants. For the CT mode, the resistance R is unchanged, $T_{t}-T_{f}$ is constant. Equation (4) then becomes:

(5) $$E^{2}=A_{\mathrm{CT}}+B_{\mathrm{CT}}\cdot \tau_{w}^{1/3}$$

where $A_{\mathrm{CT}}$ and $B_{\mathrm{CT}}$ are calibration constants in CT operation. E can be read from the oscilloscope connected to the flexible hot film shear stress sensor. In the relationship curve between $E^{2}$ and $\tau_{w}^{1/3}$, the intercept is $A_{\mathrm{CT}}$ and the slope of the curve is $B_{\mathrm{CT}}$. $B_{\mathrm{CT}}$ is called the sensitivity of the flexible hot film shear stress sensor. According to Equation (5), $B_{\mathrm{CT}}$ can be found in terms of the output voltage and the calculated shear stress [1,13].

The relationship between shear stress and the stream-wise pressure distribution can be expressed as:

(6) $$\frac{dP_{x}}{dx}=-\frac{\tau_{w}}{h}$$

where $P_{x}$ is the local pressure, in newton; h is the half height of the wind tunnel and x is the stream-wise coordinate, both reported in m [15]. Pressure taps are used to obtain the stream-wise pressure gradient. $\tau_{w}$ can be calculated by Equation (6).

**Figure 3:**
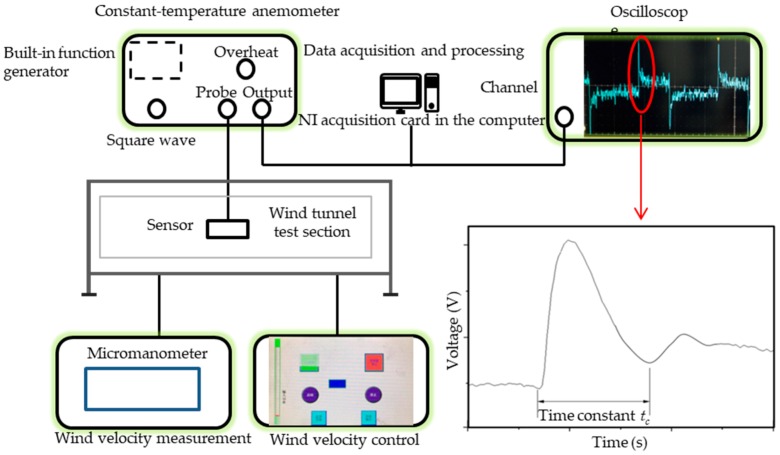
Schematic diagram of square wave and wind tunnel tests.

Time constant is used to characterize the sensor response to a step input. It is obtained by feeding square wave into the flexible hot film shear stress sensor of CT circuits. When a square wave passes through, the transient response of voltage on the thermistor is captured, from which the time constant is deduced. The time constant is determined from the experimental curve shown in Figure 3 [13]. The approximate relation between cut-off frequency $f_{c}$ and time constant $t_{c}$ is:

(7) $$f_{c}=1/t_{c}$$

## 3. Experimental Methods

The TCR tests were conducted in a constant temperature box with precision temperature control. The flexible hot film shear stress sensor was put in an incubator with wires connected to a multimeter outside. The temperature ranged from 25 °C (room temperature) to 250 °C with intervals of 10 °C. A goal temperature was set and kept for 5 min guaranteeing the uniformity and stability of the temperature in the incubator. The resistances at different temperatures were recorded, from which the TCR could be calculated. 

For the square wave tests, a constant-temperature anemometer (CTA) UNMECTA with a built-in function generator was used. The flexible hot film shear tress sensor was connected to the anemometer. A Tektronix DP05204B oscilloscope was used to graph and record the voltage change over time. The record length is 0.2 s with intervals of 0.0001 s. The record was transferred to a computer and analyzed. The schematic diagram of square wave and wind tunnel tests is shown in Figure 3. 

Wind tunnel and square wave tests were performed simultaneously. A wind velocity control was used to keep the velocity at the specific power. The wind-velocity measurement monitored the wind velocity on the flexible hot film shear stress sensor. The ambient temperature of the sensor was about 25 °C in the wind tunnel. Different wind speeds correspond to different shear stresses. An NI acquisition card was put in the computer. The computer was connected with the CTA to collect the output signal data. The calibration contained 360,000 points in a 60 s region. The flexible hot film shear stress sensor was calibrated when the output voltage at different shear stress was known.

**Figure 4:**
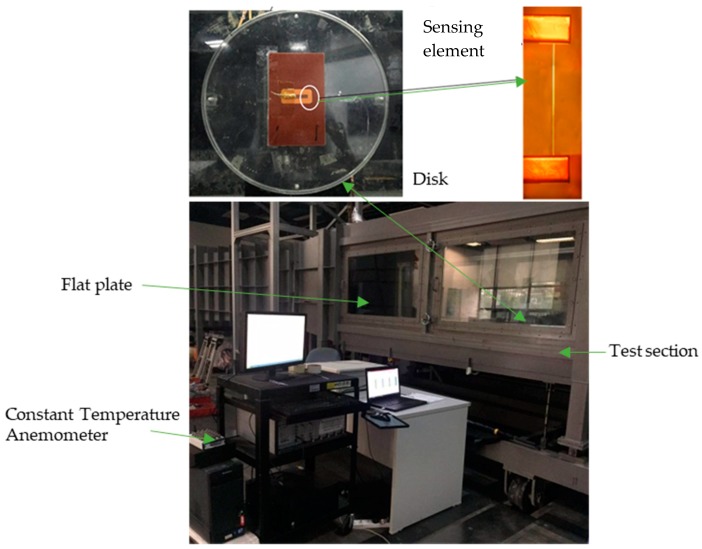
Wind tunnel setup: test section (5.6 m × 0.8 m × 1.0 m); flat plate (4.8 m × 0.78 m × 0.015 m).

Sensitivity calibration of the flexible hot film shear-stress sensor was conducted in a closed-cycle wind tunnel at the Harbin Institute of Technology, Shenzhen. Figure 4 shows the wind tunnel setup. In the test section, a flat plate was put vertically and slightly inclined to the wind direction, and was guaranteed to be a zero longitudinal pressure gradient. The flexible hot film shear stress sensor adhered to the disk was mounted on the flat plate. 

The steady-state sensor voltage was recorded at different wind velocities ranging from 2.4 to 18 m·s^−1^.

## 4. Results of Testing

There are 27 groups of flexible hot film shear stress sensors with different dimensions to be studied. Each group includes three sensors with the same dimensions. The dimensions used in the experiments are shown in Table 1.

**Figure 5:**
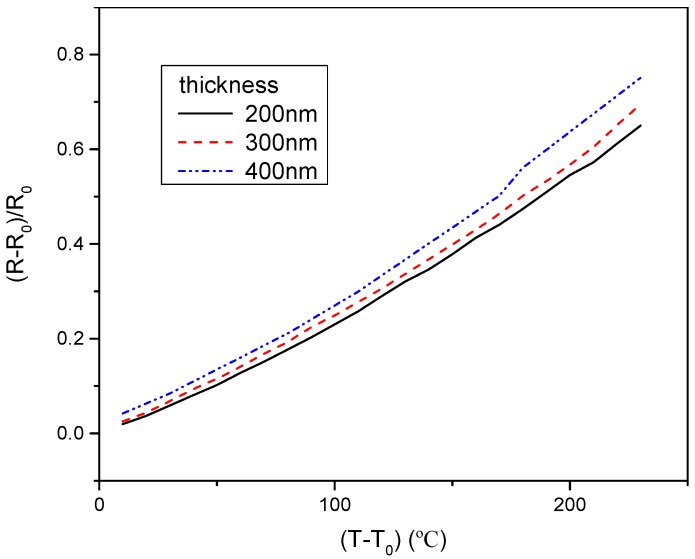
The temperature coefficient of resistance (TCR) of the flexible hot film shear stress sensor with different thickness. Estimated accuracy for (R−R_0_)/R_0_ is ±4 percent, for (T−T_0_), ±2 percent.

When the length and width of the flexible hot film shear stress sensor were unchanged, the effect of thickness on TCR was studied and the result was shown in Figure 5. It can be seen that TCR (the slope of the line) slightly increases with the increasing thickness.

Meanwhile, the TCR with different lengths and widths were studied while the other two physical dimensions were kept unchanged. The result was that the width and length had a negligible effect on the TCR of the flexible hot film shear stress sensor.

**Figure 6:**
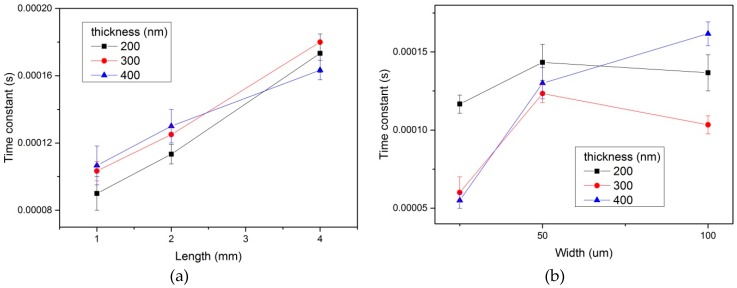
Time constant of the three groups (different thickness) of the flexible hot film shear stress sensor with different length (**a**) and width (**b**). Estimated accuracy for time constant is ±5 percent, for length and width, ±1 percent.

The results of the effect of the flexible hot film shear stress sensor dimensions (length, width, and thickness) on the time constant are shown in Figure 6. There were three groups of sensors with different thicknesses. In Figure 6a, the time constant increases with increasing length. Based on Equation (7), the frequency response decreased when the length increased. In Figure 6b, the time constant had no obvious trend when the width increased in all three groups. As shown in Figure 6a,b, the thickness of the flexible hot film shear stress sensor had no effect on the time constant.

**Figure 7:**
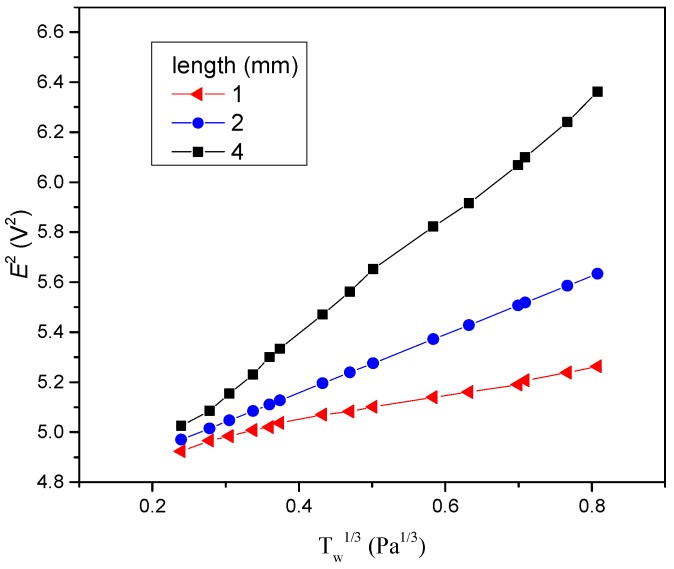
Sensitivity of the flexible hot film shear stress sensor with different lengths. Estimated accuracy for *E*^2^ is ±4 percent, for $\tau_{W}{}^{1/3}$, ±4 percent.

In Figure 7 the results of the effect of length on sensitivity of the flexible hot film shear stress sensor are demonstrated. The sensitivity is the slope of the line in Figure 7. It can be seen that when the length increased, the sensitivity increased too. In the meantime, experiments about the width and thickness effect had been conducted. The results show that the width and thickness had no significant influence on the sensitivity of the flexible hot film shear stress sensor.

## 5. Discussion of Results

The TCR of the homogeneous-distribution metal film is inversely correlated with the resistivity. The relationship between the two can be written as:

(8) αρ=constant

where ρ is the resistivity, reported in Ω·m. It is a form of Matthiessen’s rule. For continuous metal films, the conduction of electrons is affected by the scattering of the film surface, grain boundaries and defects. According to the Fuchs-Sondheimer (F-S) theory, when t>λ, the expression can be approximatively:

(9) $$\rho_{f}=\rho_{B}\left(1+3\lambda /8t\right)$$

where $\rho_{f}$ and $\rho_{B}$ are the resistivity of the film and the bulk, respectively; λ is the mean free path of electrons, and t is the film thickness, both reported in mm. The thicknesses of the hot film are all larger than ten times the mean free path of electrons of Ni. From the formula immediately above, the film resistivity $\rho_{f}$ decreases slightly with the thickness increase. Thus, the TCR increases with the film thickness increase. The experimental results are in accordance with the F-S theory. According to the formula, with increasing film thickness, the change of the TCR will be very little and can be ignored.

The frequency response increases with the decreasing length of flexible hot film shear stress sensor. The reason is that the thermal initial decreases with the decreasing length. It means that the response to the temperature change becomes faster. Thus, the frequency response increases. However, the sensitivity decreases with decreasing length. The sensing area of the flexible hot film sensor for the tested flow decreases as the length decreases, and thus so does the sensitivity. For practical applications, the equilibrium between frequency response and sensitivity should be considered to decide the length of the flexible hot film shear stress sensor. Compared with the previous work [14,15], our research clearly indicates that the flexible hot film shear stress sensor’s size influence on frequency response and sensitivity is a spanwise length effect. Experiments in our work prove that width and thickness have a negligible effect on frequency response and sensitivity.

## 6. Conclusions

The performance of flexible hot film shear stress sensors with different dimensions (length, width, and thickness) has been studied. The TCR increases with increasing thickness, but the length and width has only a minor effect on it. The length influences both the time constants (frequency response) and sensitivity. When the length grows, the sensitivity increases and the frequency response decreases. The experimental results verified that the sensitivity and the frequency response have a relationship of restricting each other. The width and thickness have no significant effect on the frequency response and sensitivity. By improving the performances through optimizing its dimensions, the flexible hot film shear stress sensor can be employed to measure the shear stress of the boundary layer instead of hot wire.

## Figures and Tables

**Table 1:** Dimensions used in the experiments.

Length (mm)	Width (μm)	Thickness (nm)
1	25	200
2	50	300
4	100	400

For example, one of the 27 groups of dimensions is 2 mm × 50 μm × 300 nm.
